# Exploring the Nexus of Token Acts of Online Support, Compassion, and Behavioral Intentions Toward Immigrants from Ukraine and Russia

**DOI:** 10.3390/bs15050564

**Published:** 2025-04-22

**Authors:** Nonna Kushnirovich, Sabina Lissitsa

**Affiliations:** 1 Ruppin Academic Center, Department of Economics and Management, Institute for Immigration and Social Integration, Emek Hefer 4025000, Israel; 2 School of Communication, Ariel University, Kiryat Hamada 3, Ariel 407000, Israel; sabinal@bezeqint.net

**Keywords:** token support, perspective-taking, empathic concerns, prosocial behavioral intentions, immigrants in Israel, asylum seekers

## Abstract

This study investigates the relationship between public token acts of online support and prosocial behavioral intentions, shedding light on the potential mediating influences that contribute to this intricate interplay. It focuses on the determinants of prosocial behavioral intentions toward three specific groups of immigrants who came to Israel after Russia’s invasion of Ukraine in 2022: asylum seekers from Ukraine, immigrant citizens repatriated from Ukraine, and immigrant citizens repatriated from Russia. The data were collected via a survey of 847 social media users in Israel. This study revealed that the higher the frequency of token acts of online support for prosocial content in social media, the higher perspective-taking and emotional concerns were reported by respondents. High emotional concern, in turn, was associated with higher prosocial behavioral intentions. Summing up, the more actively people engage with prosocial content on social media, the higher their prosocial behavioral intentions are.

## 1. Introduction

In today’s digital age, public opinions, empathy for others, and behavioral intentions are strongly affected by social media. Token acts of online support for prosocial content in social media—such as sharing, posting, and liking—may contribute to the sense of community engagement, amplify the impact of grassroots movements, foster dialogues and exchanges of ideas related to the cause, and catch the attention of decision-makers, influencers, or policymakers (Greijdanus et al., 2020). However, there is also another side to the coin. Token acts of online support, sometimes referred to as clicktivism or slacktivism, are defined as an act of willingness to support a social cause in a relatively cost-free manner, but they are also characterized by a lack of commitment to making a substantial effort to bring about meaningful change (K. Davis, 2012). In recent years, much criticism has been directed towards these acts because they are thought to stifle further prosocial behavior. Kristofferson et al. (2014) found that public token displays of support on social media result in decreased helping behavior. Similar conclusions were reached in a field experiment: despite thousands of ‘likes’, ‘shares’, and ‘comments’ on social media, only 30 users pledged any money (Lacetera et al., 2016). McKay and Dunn (2017) found that many of those who liked the Facebook page of an asylum-seeker support organization were not engaged in actions or activism around issues of asylum-seeking beyond liking the Facebook page. This phenomenon was explained by the theory of moral self-licensing, which posits that performing a prosocial action can free individuals from feeling responsible for helping because past moral behavior provides a ‘license’ to behave less morally in the present or future (Merritt et al., 2010). In contrast, Lane and Dal Cin (2018) discovered that posting a public video to social media boosted volunteers’ inclination to help. Further studies revealed that concerns about one’s moral self-image partially mediated the relationship between token support and helping behavior (Kim et al., 2023), with motivation (prosocial versus impression management) acting as a moderator (Moussaoui et al., 2022).

This study aims to delve into the underlying psychological mechanisms of prosocial behavior. Specifically, we seek to explore the role of perspective-taking and empathic concern as potential mediators in relationships between public token acts of online support for general prosocial content and prosocial behavior intentions toward immigrants, which are not connected to the subject matter of prosocial content. Analyzing these concepts in general terms allows the study to focus on universal psychological mechanisms, minimizing bias related to specific group attitudes and ensuring findings that are broadly applicable across diverse contexts and populations.

Most studies on this topic used an experimental design to examine how acts of support on social media for a specific cause, event, organization, or group relate to later prosocial behavior pertaining to that same cause or group (Kim et al., 2023; Kristofferson et al., 2014; Lacetera et al., 2016; McKay & Dunn, 2017). In contrast, little is known about the relationship between token acts of general prosocial media content support and prosocial behavioral intentions toward a specific but unrelated social group. By measuring general token acts, this study seeks to explore this broader relationship, reducing potential bias from participants’ pre-existing attitudes toward specific groups and focusing on prosocial behavioral intentions toward immigrants.

After the outbreak of the Russia–Ukraine war, many states experienced significant inflows of immigrants from these two countries. Most Ukrainian immigrants were fleeing the war. As for the newcomers from Russia, although in the beginning of the war the fighting did not take place on Russian territory, emigration for many of them was spurred by their dissent from official Russian policy. Many of them decided to emigrate out of fear of administrative penalties, criminal charges, and arrests; when Russia declared partial conscription, an additional motive for emigration emerged—to evade military mobilization and military duty (Melkumyan & Melkonyan, 2023). Thus, war was the main reason for immigration in this period.

Recent studies stressed that in Europe, the attitudes and prosocial intentions of the native population toward forced immigrants from Ukraine who came after Russia’s invasion in 2022 were more positive than attitudes and intentions toward the refugees from Africa and the East (Adamus & Grežo, 2024; De Coninck, 2023; Moise et al., 2024; Politi et al., 2023; Thérová, 2023). These studies attributed the differences in attitudes to shifts in geopolitics (Moise et al., 2024), global threats posed by the countries of origin and transferred onto forced migrants (Melkumyan & Melkonyan, 2023), and cultural similarities between them and the native population (Politi et al., 2023). However, there is a scrutiny of studies comparing prosocial behavioral intentions toward culturally similar groups of immigrants from Ukraine and Russia, who came in the same period and presented opposing sides of the same military conflict, when some of them received citizenship and others received only a temporary precarious status of group protection. This study aims to fill this gap.

Israel, like other countries, experienced a significant wave of immigration from Ukraine and Russia. In 2022, following the Russian invasion, the number of repatriates from Ukraine entering Israel increased fivefold—from 3109 in 2021 to 15,037 in 2022. The wave of repatriates from Russia was even larger, rising from 7711 in 2021 to 43,594 in 2022 (Ministry of Aliyah and Integration of Israel, 2023). Today they constitute the two largest groups of immigrants in the country. One of the reasons they chose Israel as their destination was an opportunity for some of them to receive Israeli citizenship. According to Israel’s Law of Return, Jews, their spouses, children, grandchildren, and grandchildren’s spouses are all permitted to immigrate to Israel and be granted citizenship with the status of ‘repatriates’ (Olim) (Kushnirovich, 2021). Additionally, in 2022, several tens of thousands of Ukrainians who fled the war and were not covered by the Law of Return received group protection status, and their visas are automatically extended as long as the war continues. The elderly immigrants and children up to age 18 got public health insurance, and others may apply to the national network of medical clinics “Terem”. Ukrainians who arrived following the onset of the Russia–Ukraine war were the sole non-repatriated group granted the privilege of visa extension and certain social rights in Israel. This exception was the result of the emotional response of the Israelis to the war and their empathy for forced immigrants from Ukraine (Chachashvili-Bolotin, 2023). Thus, immigration from Ukraine has led to changes in the country’s approach to accepting migrants and prosocial intentions toward them.

This study intends to provide a nuanced understanding of the factors that shape the relationship between online support and real-world prosocial behavioral intentions, and in the process shed light on potential mediating effects contributing to this interaction, comparing prosocial intentions toward the immigrants from two opposing sides of the same military conflict (Ukraine and Russia) and immigrants from the same country varying by citizen status (immigrant citizens and asylum seekers from Ukraine). This study focuses on three groups of immigrants who came to Israel after Russia’s invasion of Ukraine in 2022: asylum seekers from Ukraine, repatriated immigrant citizens from Ukraine, and repatriated immigrant citizens from Russia.

## 2. Theoretical Background and Research Hypotheses

### 2.1. Behavioral Intentions Toward Different Groups of Immigrants

The ethnic competition theory posits that rivalry for limited resources could impact attitudes toward other ethnic groups (Olzak, 1994). Since immigrants often differ from the native population of receiving countries ethnically, this theory may explain the attitudes toward them and the readiness of society to receive and support them. The feeling that one group’s welfare is at risk due to the arrival of another group and the threat posed by it results in more unfavorable outgroup emotions toward this outgroup (Scheepers et al., 2002). Two key elements affecting attitudes toward immigrants are group identification and ethnic intergroup competition for resources (Gorodzeisky & Semyonov, 2019; Kosic et al., 2023). Among those who saw immigrants as less of an economic threat, the likelihood of offering immigrants empowerment support was higher (Burhan & van Leeuwen, 2016).

Wars that begin in one country may be regarded as remote common threats by people in other countries due to the development of a “common-risk” global society (Shaw, 2017). The threat also means uncertainty. According to the uncertainty–identity theory, people who feel uncertain may choose to identify with specific social groups, form new ones, or reorganize existing ones (Hogg, 2007). In line with this theory, Kushnirovich and Lissitsa (2024) found that when global threats emerge, people may broaden the borders of their own intergroup and be more tolerant and compassionate toward the close outgroups. The social identity perspective (Olzak, 1994) holds that sentiments toward the ingroup are commonly more positive than those toward outgroups; therefore, granting people membership in the larger ingroup would result in more positive opinions toward them. People are inclined to seek affinity and proximity and express more support when faced with shared threats (Adam-Troian & Bagci, 2021).

Studies stress that the attitudes and willingness to support immigrants from Ukraine who came to Europe after Russia’s invasion in 2022 were higher than the intentions to welcome refugees from other war-torn areas of the world who came in 2015–2016, during the so-called ‘refugee crisis’ (Adamus & Grežo, 2024; Moise et al., 2024). For example, in Poland, openness and a willingness to assist Ukrainian refugees have replaced the prior reservations of the population about welcoming foreign arrivals (Thérová, 2023). Also in Slovakia, emotional reactions to the threats of war of the native Slovakians significantly improved their attitudes toward Ukrainian immigrants vs. refugees from other countries before the invasion and facilitated the willingness to help them (Adamus & Grežo, 2024). Chachashvili-Bolotin (2023) found that in Israel, immigrants who experienced the war in Ukraine only vicariously were more likely to support Ukrainians after the war started. The attitudes and willingness to support immigrants depend on their embedding in larger attitudinal patterns (especially on immigration and geopolitics) (Moise et al., 2024) and low global threats posed by Ukraine vs. high threats posed by Russia (De Coninck, 2023). Accordingly, we may hypothesize the following:

**H1.**  *There will be differences in behavioral intentions toward different groups of immigrants: immigrant repatriates from Russia, immigrant repatriates from Ukraine, and asylum seekers from Ukraine.*

### 2.2. Public Token Acts of Online Support for Prosocial Content and Prosocial Behavior Intentions

According to Smith et al. (2006), prosocial media material is any representation of prosocial conduct in words, photos, films, or interactive elements. Its goal is to arouse feelings of kindness and optimism, which will in turn lead to actions that demonstrate how these idealistic emotions are put into practice by improving society and the well-being of others (Eisenberg et al., 2013). With its emphasis on kindness, empathy, cooperation, social responsibility, and similar values, this content is intended to cultivate a sense of community, strengthen connections, and enhance the overall well-being of both individuals and groups (Coyne et al., 2018). As far as aligning with the themes of prosocial media content is concerned, participating in a cause on social media frequently represents “token support” (Kristofferson et al., 2014).

Individuals can receive social validation and recognition, especially in online communities, by participating in token actions of support on social media that give the impression of advocating desirable values. The desire to control one’s personal image and to present oneself in a positive light to others is known as self-presentation or impression management (IM) (Leary & Kowalski, 1990). Through frequent supportive reactions to prosocial content, individuals communicate their commitment to prosocial values via their social network, thus contributing to IM as people who value and promote prosocial causes. This presents the individuals in a positive light, both to themselves and to others, possibly increasing their own sense of value. Engagement in prosocial video games increased the prosocial behaviors of participants (Li & Zhang, 2023) and contributed to their perspective-taking (Nicolaidou et al., 2023).

In fact, demonstrating public token support and creating a desirable image through low-cost investment may occasionally decrease IM incentive to invest in high-cost prosocial behavior, which requires more effort than liking, signing, sharing, and posting, and sometimes may not be made public. In other words, individuals can “support” others without making any substantial investment, whether of time, energy, or money. Such low-cost online support is very attractive: it promotes a favorable public image and demands minimal commitment from the individual involved with minimal effort (Schumann & Klein, 2015). Based on the literature, we hypothesize the following:

**H2.**  *There will be a significant relationship between the frequency of public token acts of prosocial content in social media and prosocial behavior intentions toward immigrant repatriates from Russia (H2.1), immigrant repatriates from Ukraine (H2.2), and asylum seekers from Ukraine (H2.3).*

### 2.3. Perspective-Taking and Empathic Concern

Prosocial content is designed to evoke empathy and compassion. Frequent public token acts of support of prosocial content in social media and perspective-taking align with the desire for consistency, as individuals express a self-image that values prosociality and empathy. Frequent exposure to narratives, stories, or images highlighting the experiences and challenges of ‘others’, as well as active participation in supporting causes, may evoke emotional responses toward the challenging experiences of others (Hoffman, 2001).

Social Cognitive Theory suggests that individuals can develop perspective-taking and empathic concern by observing and cognitively engaging with the experiences and behaviors of others (Bandura, 2002). The taxonomy of M. H. Davis (1983) treats emphatic concern and prospective taking as independent dimensions of empathy. Empathic concern (or emotional empathy) is defined as feeling sorrow for or being concerned about others (M. H. Davis, 1983; Hoffman, 2001) and reflects an emotional element of empathy. Individuals who are actively able to tune into the thoughts and feelings of other people can vicariously experience emotions and develop empathic concern toward other people (Neumann et al., 2016; Noland, 2017).

Perspective-taking is defined as the cognitive understanding and relative awareness of other people’s emotions (often referenced as cognitive empathy) (Van der Graaff et al., 2014). Individuals who actively participate in supporting causes for disadvantaged individuals or groups are likely to develop a broader understanding of the challenges and needs they face and to adopt the spatial perspective of another person through a cognitive process. When making public token acts of online support for prosocial content, individuals’ own cognitions should be at least minimally aligned with the general public’s encouragement of prosocial acts as manifested by others. The desire to communicate a broad-minded and empathic identity to the social media audience is consonant with actively taking the viewpoint of others. It communicates to the self (and to the social network environment) that the individual is both supportive of prosocial causes and willing to understand and appreciate the experiences and viewpoints of those affected by these causes. Engaging in frequent public token acts of online support for prosocial content may activate social learning and enhance individuals’ perspective-taking abilities (Coyne et al., 2018). It may be the case even in the IM framing process, as part of constructing a desired image.

The empathy–altruism hypothesis, suggesting that individuals who experience high levels of empathy are more likely to engage in altruistic actions (Batson et al., 2007), has been a topic of interest for decades and has been supported by numerous studies (Eisenberg, 2006; Gülseven et al., 2020; Hoffman, 2001). People with a higher level of empathy tend to volunteer more, contribute more to charitable causes, and are more inclined to assist others in need, driven by genuinely altruistic motives (M. H. Davis, 1983; Grühn et al., 2008; Pang et al., 2022). A meta-analysis of studies that included 14 Western and non-Western cultures showed positive associations between empathic concern and prosocial behaviors (Miller et al., 2001). Empathic concern may lead individuals to exhibit prosocial behaviors intended to improve the well-being of various disadvantaged groups, including migrants or repatriates (Luo et al., 2024; Taylor & Glen, 2020). Perspective taking in terms of a broader understanding of the challenges and needs faced by disadvantaged individuals or groups, and taking the perspective of people or groups struggling for a better life, may also contribute to increased intention to engage in prosocial behaviors toward the deprived and poor, at least on a declarative level (Prot et al., 2014).

Based on the literature, both empathic concern and prospective taking may mediate the relationship between the frequency of public token acts of prosocial content in social media and prosocial behavior intentions. Accordingly, we may hypothesize the following:

**H3.**  *Emphatic concern will mediate the relationship between the frequency of public token acts of prosocial content in social media and prosocial behavior intentions toward immigrant repatriates from Russia (H3.1), immigrant repatriates from Ukraine (H3.2), and asylum seekers from Ukraine (H3.3).*

**H4.**  *Perspective taking will mediate the relationship between the frequency of public token acts of prosocial content in social media and prosocial behavior intentions toward immigrant repatriates from Russia (H4.1), immigrant repatriates from Ukraine (H4.2), and asylum seekers from Ukraine (H4.3).*

## 3. Materials and Methods

### 3.1. Procedure

We asked twenty seminar students studying Communications to each make a list of 30 different forums and Facebook groups on a variety of topics (politics, literature, art, travel, health, sports, hobbies, and so forth). This variety was crucial to ensure a broad and diverse pool of participants with different interests, backgrounds, and perspectives, which enhanced the representativeness of the sample and the generalizability of the findings. The students posted messages to groups asking for volunteers to be surveyed and including a link to the survey. After around 70% of the intended sample size was obtained, we made changes to our invitation letter to ensure a more representative sample that roughly matched the demographics of Israeli Internet users.

The target sample size was determined based on Preacher and Hayes (2004). A minimum of 500 participants was estimated to provide sufficient statistical power (0.80) for effects (Faul et al., 2007). To ensure representativeness and allow for potential exclusions, we aimed for a larger sample. Respondents’ personal information, like email addresses and phone numbers, was not included in the anonymous poll.

### 3.2. Sample

Data were collected via a survey of 847 social media users in Israel. The mean age of the sample was 28.44 (*SD* = 6.33), corresponding to national statistics, which indicated that most Israeli social media users were 25–34 years old (Statista, 2022). A total of 51.4 percent of the respondents were female, and 16.3 percent were foreign-born immigrants (mean length of time living in Israel was 21.5 years (*SD* = 7.91)), which corresponds to national statistical data for the Israeli population.

### 3.3. Measurements

Dependent variables. Behavioral intentions toward immigrant repatriates from Russia/immigrant repatriates from Ukraine/asylum seekers from Ukraine were based on the index developed by Mancini et al. (2020) and were formulated by the following question: “To what extent are you ready or not ready to assist in the following ways: host them at your home for a few days; donate an essential (for you) sum of money to associations that help this group; donate clothes, shoes, or household items to them; write them a message that supports and lifts the spirit; give a little help (e.g., help them to communicate with the authorities, help with children, carry things, etc.). The items were scaled from 1 to 5. Three dependent index variables were calculated as mean values of these five items. Internal reliability for all the indices was good: Cronbach’s Alpha for immigrant repatriates from Russia was 0.837; for immigrant repatriates from Ukraine, 0.828; and for asylum seekers from Ukraine, 0.815. The validity of the indices was also justified by confirmatory factor analysis (CFA) that included three latent variables of behavioral intentions toward three different groups, and showed good fit indices: *CFI* = 0.985, *IFI* = 0.985, *TLI* = 0.976, *RMSEA* = 0.063, and *p* < 0.001.

Independent variables. The frequency of public token acts of support of prosocial content in social media was based on Noland’s (2017) Slacktivism Engagement scale and was tested by the following item: when you are exposed to content in which people help each other, volunteer for the community or disadvantaged groups, donate, support and encourage each other on social media, how often do you engage in the following activities: (1) reply with a ‘like’ or ‘smile’ emoji; (2) share things that interest me; (3) write comments on things other people posted; (4) write my own posts (statuses) as a response to what I was exposed to; and (5) upload pictures or videos in response to what I was exposed to. The answers were on a scale of 1 (not at all) to 5 (very high frequency); the internal reliability of the index was good (*Cronbach’s Alpha* = 0.847).

Mediators. The mediator variables perspective-taking (5 items, scaled 1–5) and emphatic concern (4 items, scaled 1–5) were based on dimensions of the Interpersonal Reactivity Index (M. H. Davis, 1983). Although M. H. Davis’s (1983) constructs included 7 items for each dimension, we omitted items with low factor loadings in our sample. The validity of these indices was justified by CFA, which included two latent variables of perspective-taking and empathic concern (*CFI* = 0.981, *IFI* = 0.981, *TLI* = 0.974, *RMSEA* = 0.051, and *p* < 0.001). Both indices showed good internal reliability (*Cronbach’s Alphas* were 0.849 and 0.790, respectively). Definitions and descriptions of the variables are presented in Table 1.

## 4. Results

### 4.1. Descriptive Statistics and Differences in Behavioral Intentions

The frequency of public token acts of online support for prosocial content in social media reported in our sample was rare, *M* = 2.44 (*SD* = 1.14) on a scale of 1–5. Most frequently, respondents put a “like” or another smile item *M* = 3.32 (*SD* = 1.56), and most rarely they uploaded their own content: wrote posts (*M* = 1.93, *SD* = 1.32) or uploaded pictures or videos (*M* = 1.96, *SD* = 1.35). The levels of reported perspective-taking and empathic concern were higher than the center of the scale (*M* = 3.76, *SD* = 0.87 and *M* = 3.95, *SD* = 0.83 on a scale of 1–5, correspondingly).

To compare the levels of behavioral intentions toward the three groups of immigrants, we ran the Friedman test, which showed significant differences between the intentions toward these groups (*χ*^2^(2) = 38.779, *p* < 0.001). Thus, H1 which hypothesized there would be differences between the groups, was supported. The pairwise comparison showed that the behavioral intentions toward immigrant repatriates from Ukraine were significantly higher (*M* = 3.32, *SD* = 1.01) than the intentions toward immigrant repatriates from Russia (*M* = 3.22, *SD* = 1.03), *z* = 4.332, *p* < 0.001. No differences were found between behavioral intentions toward immigrant repatriates from Ukraine and behavioral intentions toward immigrant asylum seekers from Ukraine (*M* = 3.26, *SD* = 1.00), *z* = 2.274, *p* = 0.069, and between behavioral intentions toward immigrant repatriates from Russia and behavioral intentions toward immigrant asylum seekers from Ukraine, *z* = 2.058, *p* = 0.119.

### 4.2. Testing the Relationships Between Token Acts, Empathy, and Behavioral Prosocial Intentions

**Figure 1:**
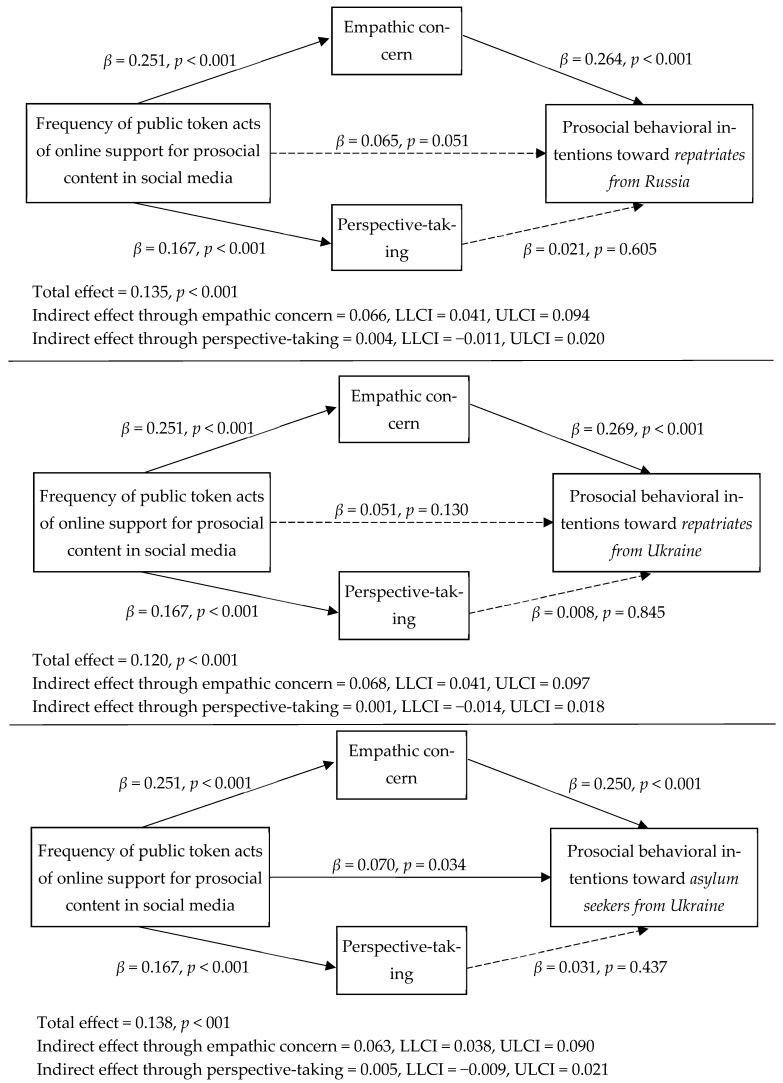
Standardized effects of regressions.

To examine the relationships, we used PROCESS Procedure, Version 4.2 for SPSS as proposed by Preacher and Hayes (2004).^1^ Model 4 was run separately for three dependent variables: behavioral intentions toward immigrant repatriates from Russia, toward immigrant repatriates from Ukraine, and toward asylum seekers from Ukraine. In the models, we also controlled for sex, age, education, household income, political views, and being an immigrant, including them as independent variables. The direct, indirect, and total standardized effects are reported in Figure 1.

The study found a positive total effect of the frequency of public token acts of online support for prosocial content in social media on prosocial behavior intentions toward all three groups of immigrants: immigrant repatriates from Russia (*β* = 0.135, *p* < 0.001), immigrant repatriates from Ukraine (*β* = 0.120, *p* < 0.001), and asylum seekers from Ukraine (*β* = 0.138, *p* < 0.001). Hypotheses H2.1, H2.2, and H2.3 were supported.

This study found a positive relationship between the frequency of public token acts of online support for prosocial content in social media and emphatic concern (*β* = 0.251, *p* < 0.001). It also found positive relationships between empathic concern and prosocial behavioral intentions toward three groups of immigrants: repatriates from Russia (*β* = 0.264, *p* < 0.001), repatriates from Ukraine (*β* = 0.269, *p* < 0.001), and asylum seekers from Ukraine (*β* = 0.250, *p* < 0.001). The indirect effects through empathic concern were deemed significant (with a 95% confidence interval not including 0) for all three groups of immigrants (*β* = 0.066 [0.041; 0.094] for immigrant repatriates from Russia, *β* = 0.068 [0.041; 0.097] for immigrant repatriates from Ukraine, and *β* = 0.063 [0.038; 0.090] for asylum seekers from Ukraine. Thus, empathic concern was a mediator of the relationship between the frequency of public token acts of support of prosocial content in social media and prosocial behavioral intentions toward immigrants; hypotheses H3.1, H3.2, and H3.3 were supported. For immigrant repatriates from Russia and immigrant repatriates from Ukraine the mediation was full since the direct effects of frequency of public token acts of online support for prosocial content in social media on prosocial behavioral intentions after mediation were non-significant (*β* = 0.065, *p* = 0.051 and *β* = 0.051, *p* = 0.130, correspondingly). For asylum seekers from Ukraine, the direct effect after mediation was significant (*β* = 0.070, *p* = 0.034), namely, there was only partial mediation for them.

There was a positive relationship between the frequency of public token acts of support of prosocial content in social media and perspective-taking (*β* = 0.167, *p* < 0.001). However, no significant relationships were found between perspective-taking and prosocial behavior intentions toward immigrant repatriates from Russia (*β* = 0.021, *p* = 0.605), immigrant repatriates from Ukraine (*β* = 0.008, *p* = 0.845), and asylum seekers from Ukraine (*β* = 0.031, *p* = 0.437). Thus, perspective-taking did not mediate the relationship between the frequency of public token acts of support of prosocial content in social media and prosocial behavioral intentions toward immigrants; hypotheses H4.1, H4.2, and H4.3 were not supported.

We also found a significant relationship between political views and prosocial behavioral intentions toward immigrants; more left-wing views were associated with higher prosocial behavioral intentions toward all the groups of immigrants (*β* = 0.127, *p* < 0.001 for immigrant repatriates from Russia, *β* = 0.104, *p* = 0.002 for immigrant repatriates from Ukraine, and *β* = 0.146, *p* < 0.001 for asylum seekers from Ukraine). Men had lower prosocial behavioral intentions than women (*β* = −0.086, *p* = 0.011 for immigrant repatriates from Ukraine, and *β* = −0.102, *p* < 0.002 for asylum seekers from Ukraine). The effects of age, education, household income, and being an immigrant on prosocial behavioral intentions toward all the groups were non-significant.

## 5. Discussion

This study aimed to address the need for a more nuanced knowledge of the mechanisms influencing the relationship between online support and in-person prosocial activities with a focus on potential mediating elements that may contribute to this complex interplay. It explained the complex dynamics of token online support for prosocial content via actions like liking, posting, and sharing, wherein public displays have been found to be associated with prosocial behavior intentions. In our study, we revealed the determinants of prosocial behavior intentions toward asylum seekers from Ukraine, immigrant citizens from Ukraine, and immigrant citizens from Russia who came to Israel after Russia’s invasion of Ukraine in 2022, groups that were not connected to the subject matter of the examined prosocial online content. This study also explained the psychological mechanisms of this phenomenon, finding that empathic concern mediated the relationship between frequent public token displays of prosocial content in social media and prosocial behavior intentions. This provides a more thorough framework for future research into the psychology of online prosocial behavior.

This study found that behavioral intentions toward immigrant repatriates from Ukraine were higher than those toward immigrant repatriates from Russia, and not significantly different from behavioral intentions toward asylum seekers from Ukraine. It seems that the sentiment and compassion toward repatriates from Ukraine were more essential than toward repatriates from Russia. The status of being a citizen or asylum seeker did not matter. One possible explanation is that, in line with Moise et al. (2024), the intentions of the native population to help immigrants depend on broader patterns of attitudes, particularly on immigration and geopolitics, as well as on global threats posed by the country of immigrants’ origin. If previous studies attributed different attitudes to different groups of forced migrants who came in different periods (forced migrants from Ukraine since 2022 vs. refugees from Africa and the East since 2015) to changing geopolitics and cultural similarities between them and the native population, this study found that even if immigrants fled their countries of origin because of the same war, the prosocial behavioral intentions toward them were different. It seems that immigrants who come from countries representing the ‘wrong’ side of the conflict from the local population’s point of view may be treated differently. The relationships to the countries of origin, because of global threats posed by them and not supporting their policy, may be carried out to immigrants as their representatives, even though they left because of disagreement with government policy, and sometimes are even at risk of persecution in their countries of origin.

This study revealed that the higher the frequency of public token acts of online support for general prosocial content in social media was, the more prosocial behavioral intentions were reported toward different types of immigrants. The theories of moral self-licensing (Merritt et al., 2010) and the slacktivism effect described by Kristofferson et al. (2014), claiming that public token displays of support on social media result in decreased helping behavior, were not supported. One possible explanation is that *clicktivism or slacktivism* (relatively costless token displays of support with low willingness to act for essential change) occurs when public online support and prosocial behavior intentions relate to the same kind of activity and happen in sequence. Online support creates in the individual the feeling that s/he has already done something, and there is no need to do anything else, and virtual activity substitutes for real action to improve the situation Kristofferson et al. (2014). But when we talk about the support of general prosocial content, a feeling of having already done something may not arise, and as such, the substitution effect does not occur. On the contrary, people who actively demonstrate online support for any prosocial content are more likely to be inclined to any other prosocial activity and have prosocial behavior intentions. This phenomenon occurs only under the condition that the subject matter of prosocial content on social media does not connect to the subject matter of prosocial activity.

This study revealed that the higher the frequency of public token acts of online support for prosocial content in social media, the higher both dimensions of empathy: perspective-taking and emotional concerns, were reported by respondents. When taking a stance through publicly tokenized online support for prosocial material, individuals’ own thoughts ought to correspond, at least minimally, with the public endorsement of prosocial actions by others. Actively accepting other people’s points of view is in consonance with the goal of posting tolerant material for the social media community. It sends a message to one’s inner circle and social network that the person not only supports these prosocial issues but also comprehends and appreciates the perspectives and experiences of others impacted by them. The goal of prosocial content is to arouse sympathy and empathy. People who exhibit a prosocial and empathic self-image are more likely to engage in frequent public token acts of support of prosocial content on social media and perspective-taking, which are consistent with the desire to maintain consistency and to elicit empathetic reactions.

This study found that the positive relationship between public token acts of online support for prosocial content in social media and prosocial behavioral intentions was mediated by empathic concerns. Frequently performing online token actions of public support for prosocial content stimulates emotional reactions such as empathic concern and sincere care for other people’s welfare. In turn, people with high levels of empathy are more inclined to act altruistically (Batson et al., 2007). Those who exhibit empathy for others may choose to take prosocial actions to better the lives of various marginalized groups, such as migrants or repatriates (Prot et al., 2014; Taylor & Glen, 2020). In essence, an emotional bond boosts the desire to act prosocially toward impoverished and destitute populations, like newcomers who are usually disadvantaged in a new country, at least until they have acclimatized.

Perspective-taking or consciously tuning into other people’s thoughts and feelings, and taking on their spatial perspective, did not foster prosocial behavioral intentions. It seems that cognitive processes did not affect prosocial behavior as much as the emotional process did. We can conclude that the emotional process is more important for fostering the mechanism of prosocial behavior than the cognitive process of perspective-taking.

## 6. Conclusions

In summation, public token acts of support for general prosocial content produce stronger prosocial behavioral intentions, mediated by emotional concerns. Crucially, this study draws a line between generic prosocial content and behavioral intentions. The potential for social media to foster true empathy and compassion is shown by the positive association between activities taken online and prosocial conduct in the real world, especially when it comes to meeting the needs of disadvantaged groups.

These findings also suggest that promoting general prosocial content on social media, unrelated to specific causes, may be a powerful catalyst for authentic engagement and support across a wide range of social issues, offering a way to create positive social impact through digital platforms. Organizations and governments can use the findings to build social media interventions that promote empathy, improving the likelihood of meaningful prosocial activity in varied social circumstances.

### Study Limitations

This study examined prosocial behavior intentions toward three specific groups of immigrants in only one country; further cross-country analysis is needed. Although the use of general measures of token acts of public support, emphatic concern, and perspective-taking provides insights into overarching prosocial tendencies, it does not capture group-specific nuances, such as empathy directed specifically toward refugees from the Russian–Ukrainian conflict. Future research could explore whether these general patterns vary when specific groups or contexts are explicitly mentioned.

While this study provides insights into the psychological mechanisms underlying prosocial token acts and intentions, the use of self-reported intentions as an outcome measure limits the ability to directly infer actual helping behaviors. Measuring actual prosocial behaviors toward refugees presents significant methodological challenges, as direct observation is often impractical and self-reported behaviors may be influenced by recall or social desirability biases. Future research should aim to incorporate observational or experimental methodologies to bridge the gap between intentions and real-world behaviors. Another limitation is that only two mediators were examined: perspective-taking and emotional concerns. Further studies should focus on other mediators.

## Figures and Tables

**Table 1:** Variables description.

Variable	Definition	*Mean*	(*SD*)	%
1. Frequency of public token acts of online support for prosocial content in social media	Based on 5 items scaled 1–5, from 1 = never, to 5 = very often (*Cronbach’s Alpha* = 0.847):	2.44	(1.14)	
	1.1. Make a “like” or another smile	3.32	(1.56)	
	1.2. Share things that interest me	2.64	(1.53)	
	1.3. Write comments on things other people posted	2.38	(1.45)	
	1.4. Write my own posts (statuses) as a response to what I was exposed to	1.93	(1.32)	
	1.5. Upload pictures or videos in response to what I was exposed to	1.96	(1.35)	
2. Perspective-taking	Based on 5 items scaled 1–5, from 1 = do not agree at all, to 5 = agree to a high extent (*Cronbach’s Alpha* = 0.849):	3.76	(0.87)	
	2.1. Before I criticize someone, I try to imagine how I would feel in his/her place	3.73	(1.14)	
	2.2. Sometimes I imagine how things look from the point of view of others to understand them better	3.80	(1.08)	
	2.3. I believe there are two sides to a coin, and I try to look at both	3.99	(1.04)	
	2.4. When there is a disagreement between two people, I try to understand each one’s reasons	3.90	(1.07)	
	2.5. When I am angry with someone, I usually try to “put myself in their shoes” for a while	3.37	(1.18)	
3. Empathic concern	Based on 4 items scaled 1–5, from 1 = do not agree at all, to 5 = agree to a high extent (*Cronbach’s Alpha* = 0.790):	3.95	(0.83)	
	3.1. When I see someone being taken advantage of, I feel the need to protect him/her	4.17	(1.02)	
	3.2. I feel affection and care for the people who are less fortunate than me	3.66	(1.14)	
	3.3. I describe myself as a rather soft-hearted person	3.90	(1.03)	
	3.4. I really care about the things that happen around me	4.07	(1.02)	
4. Behavioral intentions toward immigrant repatriates from Russia	Based on 5 items scaled 1–5, from 1 = I am not ready at all, to 5 = sure I am ready (*Cronbach’s Alpha* = 0.837):	3.22	(1.03)	
	4.1. Host them at your house for a few days	2.43	(1.35)	
	4.2. Donate an essential (for you) sum of money to associations that help new immigrant repatriates from Russia	2.75	(1.29)	
	4.3. Donate clothes, shoes, or household items to them	3.77	(1.29)	
	4.4. Write them a message that supports and lifts their spirit	3.58	(1.38)	
	4.5. Give a little help (e.g., help them to communicate with the authorities, help with children, carry things, etc.)	3.56	(1.32)	
5. Behavioral intentions toward immigrant repatriates from Ukraine	Based on 5 items scaled 1–5, from 1 = I am not ready at all, to 5 = sure I am ready (*Cronbach’s Alpha* = 0.828):	3.32	(1.01)	
	5.1. Host them at your house for a few days	2.50	(1.36)	
	5.2. Donate an essential (for you) sum of money to associations that help new immigrant repatriates from Ukraine	2.85	(1.32)	
	5.3. Donate clothes, shoes, or household items to them	3.90	(1.25)	
	5.4. Write them a message that supports and lifts their spirit	3.70	(1.35)	
	5.5. Give a little help (e.g., help them to communicate with the authorities, help with children, carry things, etc.)	3.65	(1.31)	
6. Behavioral intentions toward asylum seekers from Ukraine	Based on 5 items scaled 1–5, from 1 = I am not ready at all, to 5 = sure I am ready (*Cronbach’s Alpha* = 0.815):	3.26	(1.00)	
	6.1. Host them at your house for a few days	2.40	(1.34)	
	6.2. Donate an essential (for you) sum of money to associations that help new immigrant repatriates from Ukraine	2.75	(1.33)	
	6.3. Donate clothes, shoes, or household items to them	3.85	(1.28)	
	6.4. Write them a message that supports and lifts their spirit	3.69	(1.36)	
	6.5. Give a little help (e.g., help them to communicate with the authorities, help with children, carry things, etc.)	3.58	(1.32)	
Age	Continuous variable, years	28.44	(6.33)	
Education	1 = less than high school;	3.93	(1.30)	
	2 = high school			18.8
	3 = vocational studies			15.6
	4 = student for undergraduate degree			28.5
	5 = undergraduate degree			26.1
	6 = graduate degree or higher			11.0
Income of the household	1 = no income	7.46	(2.98)	7.1
	2 = less than 2000 NIS			4.3
	3 = 2001–3000			4.3
	4 = 3001–4000			4.3
	5 = 4001–5000			4.7
	6 = 5001–6000			4.9
	7 = 6001–7500			7.3
	8 = 7501–10,000			16.4
	9 = 10,001–14,000			16.4
	10 = 14,001–21,000			15.3
	11 = more than 21,000 NIS			14.9
Political views	Scaled 1–5, from 1 = right wing to 5 = left wing	2.48	(0.87)	
Being an immigrant				16.3

## Data Availability

Restrictions apply to the availability of these data. Informed consent included the statement that the data would only be accessible to authorized project researchers.

## Note

1 We also ran alternative models in which token online support for prosocial content mediated the relationship between perspective-taking, emotional concerns, and behavioral intentions. The percent of variance in prosocial behavioral intentions explained by these models was lower. We decided not to report the detailed results because of the limited length of the paper.
